# Exploring Morphometry of Endometrial Glands and Blood Vessels in Reproductive Age Women With Abnormal Uterine Bleeding

**DOI:** 10.7759/cureus.70303

**Published:** 2024-09-27

**Authors:** Muni Bhavani Itha, Ramya Katta, Golla Reethi S Grace, Aparna Chinam, Kakumanu Nageswara Rao

**Affiliations:** 1 Pathology, Guntur Medical College, Guntur, IND

**Keywords:** angiogenesis, endometrium, height of glandular epithelium, mean vascular density, mitotic score, vascular diameter

## Abstract

Background

Abnormal uterine bleeding (AUB) is one of the most common problems encountered in gynaecological practice. Defective endometrial angiogenesis has been implicated in various benign and malignant disorders of the endometrium.

Aim

This study aims to assess morphometry of endometrial glands and blood vessels in patients with AUB.

Material and methods

This is a one year retrospective cross sectional analysis of endometrial samples received with the diagnosis of AUB in reproductive age group. All samples were routinely processed and stained. Sections were analysed for morphometry of blood vessels and endometrial glands.

Results

A total of 374 cases were included. Most common histological group was proliferative followed by secretory phase. A significant difference was noted in mean vascular density, diameter, mitotic scores and height of glandular epithelium in different benign and malignant groups.

Conclusion

This study highlights the fact that glandular and vascular morphometry can be used to differentiate between various proliferative disorders of the endometrium. In the current era of new anti-angiogenic therapies, endometrial angiogenesis and changes in vascular morphology can be targeted, thus improving treatment modalities and patient care.

## Introduction

Any bleeding from the uterine corpus that is abnormal in terms of frequency, regularity, duration, or volume of flow in the absence of pregnancy is considered abnormal uterine bleeding (AUB), according to the International Federation of Gynaecology and Obstetrics [1]. AUB can manifest either as heavy menstrual bleeding or inter-menstrual bleeding. Most often, heavy menstrual bleeding is associated with structural abnormalities like fibroids or polyps, and inter-menstrual bleeding is associated with ovulatory dysfunction or endometrial disorders. AUB affects about 14-25% of women in the reproductive age group, and this may have a significant impact on their physical, emotional, and social well-being [2]. Radiological examination, being a non-invasive method, remains the first choice of investigation in most cases. However, endometrial sampling and examination under a microscope are quintessential in cases with no anatomic abnormalities. Understanding the pathogenesis and mechanisms of these disorders is still poor, and thus, the development of non-surgical therapies is still lacking.

The human endometrium is a distinctive organ that undergoes cyclical regeneration through the remodeling and restoration of endometrial blood vessels, endometrial glands [3], and stroma. Aberrations in angiogenesis are advocated to be an important cause of AUB. Thus, endometrial blood vessels and endometrial gland morphometry could be a useful parameter for distinguishing different causes of AUB. The present study was taken up with an aim to assess the histopathology and morphometry of endometrial gland and blood vessel in different causes of AUB and also to analyse if there exists a significant difference in these measurements in various lesions of endometrium.

## Materials and methods

The present study is a one-year retrospective, cross-sectional, analytical study conducted in the Department of Pathology in a tertiary care teaching hospital in South India. Cases received between September 2022 and August 2023 were retrieved from departmental archives and analyzed retrospectively after obtaining ethical clearance from the Institutional Ethics Committee, Guntur Medical College and Government General Hospital, Guntur (approval number: GMC/IEC/20/2023).

Inclusion criteria: All the patients belonging to reproductive age group (15 to 49 years) diagnosed as AUB due to primary endometrial pathology for whom endometrial sampling (including dilatation and curettage, biopsies, fractional curettage and hysterectomy specimens) was performed were included in the study.

Exclusion criteria: Cases from the post-menopausal age group, cases on hormonal therapy, patients diagnosed as AUB due to cervical, myometrial, or vaginal causes and AUB due to coagulopathy, patients with a history of miscarriages, patients who underwent repeat biopsy after any kind of treatment were excluded.

Sample size calculation: The estimated minimum sample size required, with incidence rate of AUB in reproductive age group as 14 and 25%, with 95% confidence interval and 5% precision was 186 and 289 cases respectively. A total of 374 cases were included in the present study by convenience sampling.

Method: The endometrial samples thus received were routinely fixed in 10% neutral buffered formalin, processed using automatic tissue processor Leica TP1020 (Leica Biosystems, CA), paraffin-embedded, sectioned manually, and stained routinely with hematoxylin and eosin. Morphometric analysis was performed using Olympus CX21i LED bright-field trinocular microscope (Olympus Corporation, Japan) with a field number of 20, field diameter of 0.5mm, and field area of 0.196mm^2^ in 400X magnification. Magnus Mag cam HD pro (6 megapixel) HDMI CMOS digital camera (Magnus Opto Systems India Pvt. Ltd., India) was used for morphometric analysis.

All the cases were analysed for shape of glands (Tubular, Coiled, Ramified, Papillary, Cylindrical), gland: stroma ratio (normal or increased), presence of vascular dilatation, presence of vascular congestion, presence of stromal hemorrhages, mean microvascular density (number of blood vessels per mm^2^), mean vascular diameter (in µm), height of glandular epithelium (in µm) and number of mitotic figure per mm^2^. In the present study, a histological feature was considered to be striking when it was found in atleast 70% of the cases in each group included in the study. For calculating vascular diameter and height of glandular epithelium, a minimum of five 400X fields (approximately 1mm^2^ area) were taken into consideration, and all the glands or vessels in that field were measured, and a mean of the values thus obtained was calculated. Mitotic index was designated as 1+ for 0-5 mitotic figures per 2mm^2^, 2+ for 6-10 mitotic figures per 2mm^2^, 3+ for 10-15 mitotic figures per 2mm^2^, 4+ for 15-20 mitotic figures per 2mm^2^ and 5+ for > 20 mitotic figures per 2mm^2^. Height of glandular epithelium was calculated as half of the difference between glandular and luminal diameter. At least 20 non disrupted glands were counted and mean was obtained.

Statistical analysis: Results were tabulated in Microsoft Excel 2016 version. Frequencies and percentages were calculated for all categorical data and means were calculated for continuous variables. Statistical Package for the Social Sciences (SPSS) Software, version 24 (IBM Corp., Armonk, NY) was used for statistics. Value of significance was calculated using independent T test and p value less than or equal to 0.05 was considered significant.

## Results

A total of 374 cases were included in the study. The mean age of the patients included in the study was 41.54±8.46 years, with the most common age group in the 4th and 5th decades of life. In the present study, the proliferative phase of the endometrium (45.5% of all cases) was the most common histopathological diagnosis, followed by secretory phase endometrium (31.0% of all cases) and the least common diagnosis was endometrial hyperplasia with atypia (2.9%) closely followed by endometrial carcinomas (3.1%) (Table 1).

**Table 1:** The number of cases and average age of all the cases included in the present study PP: Proliferative phase, SP: Secretory phase, EP: Endometrial polyp, DPP: Disordered proliferative phase, HA: Hyperplasia without atypia, EIN: Endometrial intra epithelial neoplasia, EC: Endometrial carcinoma.

Clinical details	PP	SP	DPP	EP	HA	EIN	EC
Number of cases (n=374)	170	116	19	20	25	11	13
Percentage of total(%)	45.5	31.0	5.1	5.3	6.7	2.9	3.5
Average age (years) (Mean±SD)	39.78± 6.29	38.78± 6.19	41.53± 4.45	41.95± 5.31	43.96± 5.53	39.73± 6.50	45.23± 3.79

**Figure 1:**
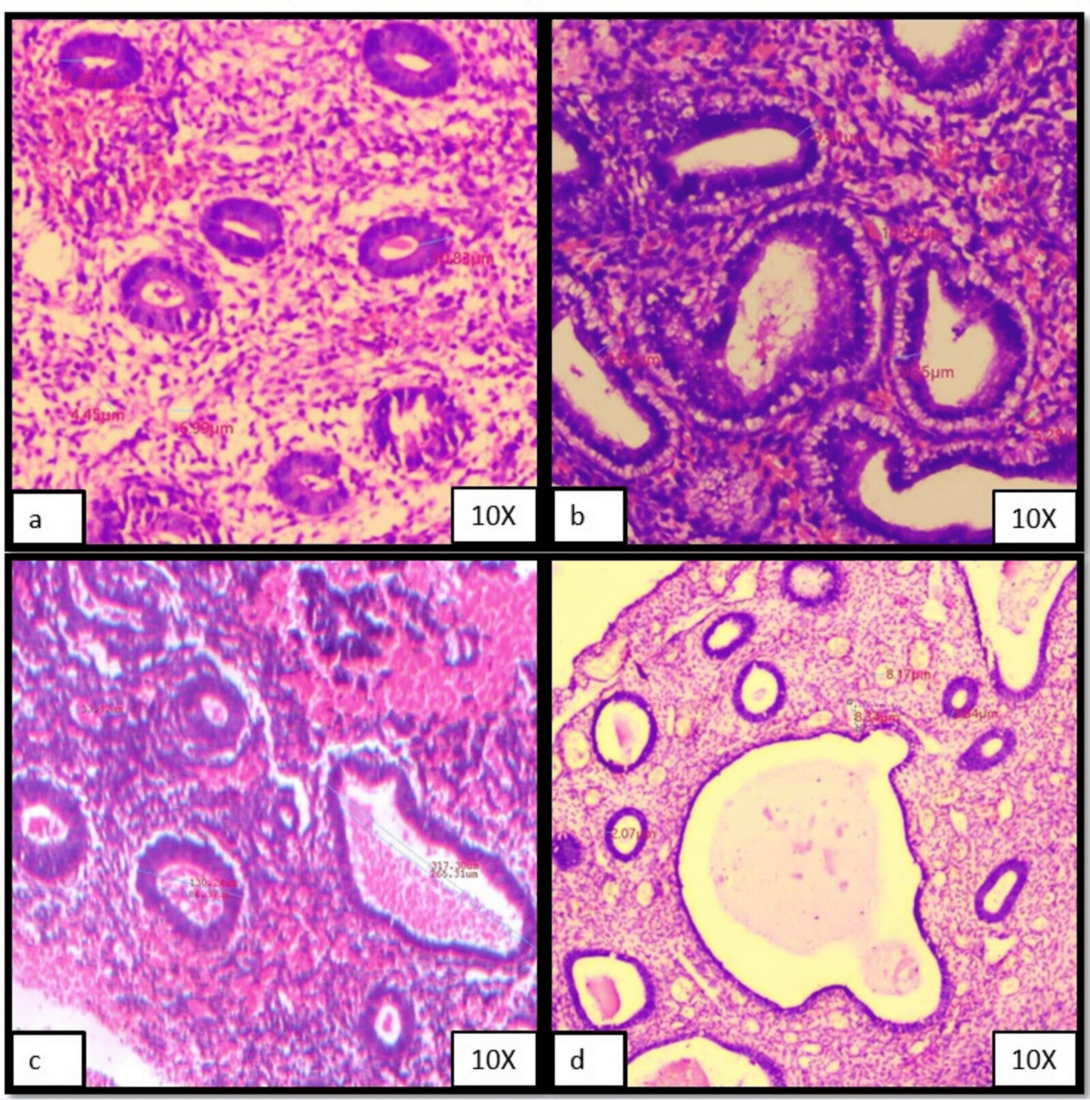
Photomicrographs showing morphometric analysis results (set I) (a) Proliferative endometrium, (b) Secretory phase, (c) Disordered proliferative endometrium, (d) Endometrial polyp.

**Figure 2:**
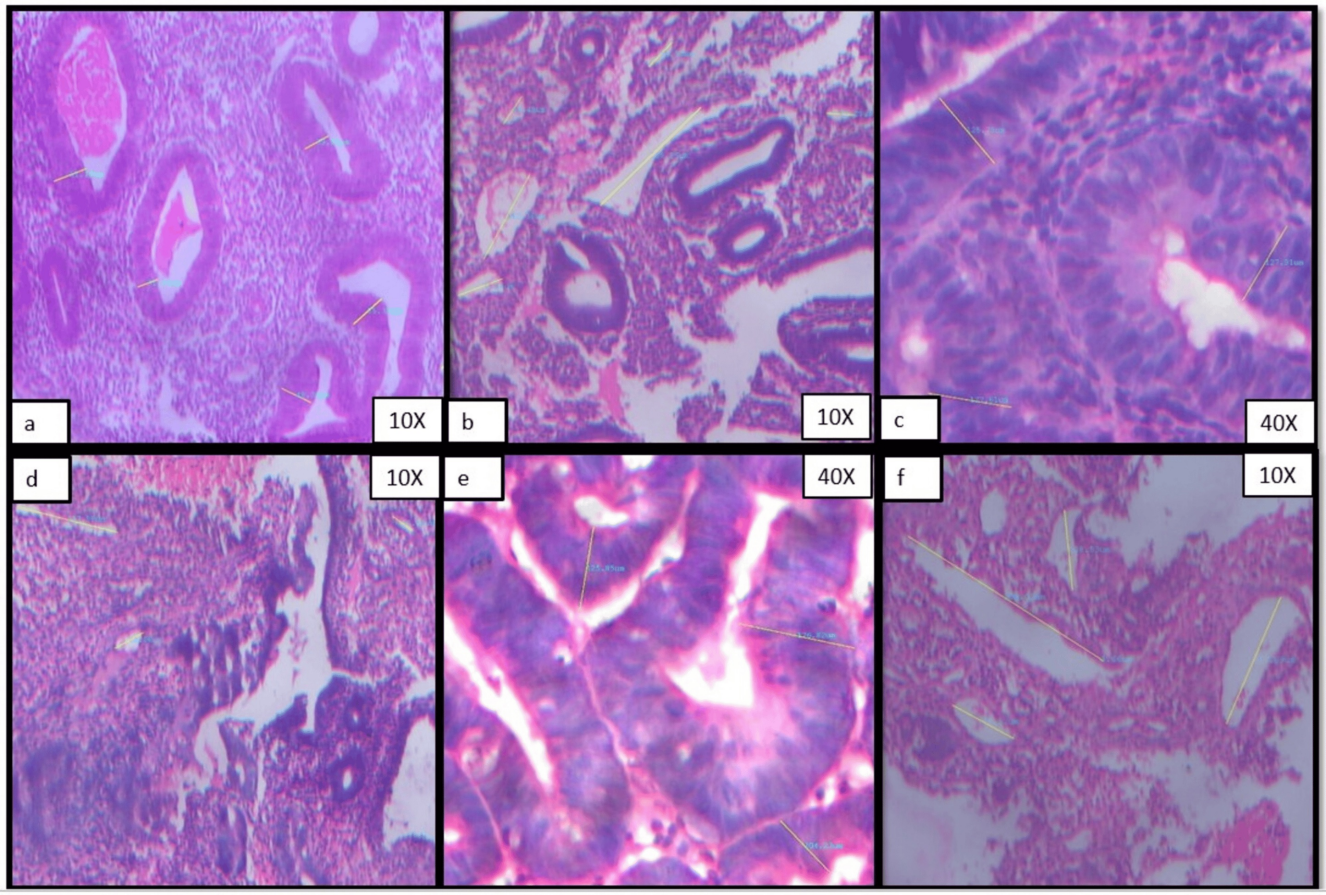
Photomicrograph showing morphometric analysis (set II) (a) Height of glandular epithelium in a case of hyperplasia without atypia, (b) Vascular diameter in a case of hyperplasia without atypia, (c) Height of glandular epithelium in a case of endometrial intraepithelial neoplasia, (d) Vascular diameter in a case of endometrial intraepithelial neoplasia, (e) Height of glandular epithelium in a case of endometrial carcinoma, (f) Vascular diameter in a case of endometrial carcinoma.

Figures 1a-1d and Figures 2a-2f show representative cases of morphometric features of various histological groups that were included in the present study.

Table 2 depicts the distribution of all cases based on the shape of the gland, gland-to-stroma ratio, presence of vascular congestion, vascular dilation, presence of stromal hemorrhages, and mitotic scores in each histopathological group. As highlighted in Table 1, salient features in proliferative phase cases were tubular glands, normal gland-to-stroma ratio, absence of vascular dilation, congestion, and stromal hemorrhages with low mitotic score (score 1), whereas in secretory phase cases, salient features observed included coiled glands with increased gland to stroma ratio, presence of vascular congestion and dilation, absence of stromal hemorrhages and low mitotic score (score 1). Salient features of disordered proliferative endometrium in the present study were increased gland-to-stromal ratio, vascular dilation, and low mitotic score. Endometrial polyps showed prominent vascular dilation, congestion, and low mitotic score. In cases of endometrial epithelial tumor precursor lesions, both endometrial hyperplasia without atypia and endometrial intraepithelial neoplasia showed increased gland-to-stroma ratio as the most salient feature. Moreover, endometrial intraepithelial neoplasia showed ramified glands, the presence of vascular congestion, dilation, stromal hemorrhages, and a higher mitotic score (score 3) in most cases. The salient features of Endometrial carcinomas were increased gland to stroma ratio and high mitotic score (score 4).

**Table 2:** The distribution of cases in various histological groups based on different morphological parameters PP: Proliferative phase, SP: Secretory phase, EP: Endometrial polyp, DPP: Disordered proliferative phase, HA: Hyperplasia without atypia, EIN: Endometrial intra epithelial neoplasia, EC: Endometrial carcinoma.

Histological features		PP (%)	SP (%)	DPP (%)	EP (%)	HA (%)	EIN (%)	EC (%)
Shape of gland	Tubular	120(70.6)	12(10.3)	0(0)	0(0)	0(0)	0(0)	0(0)
	Coiled	48(28.2)	101(87.1)	0(0)	0(0)	0(0)	0(0)	1(7.7)
	Ramified	2(1.2)	3(2.6)	10(52.6)	10(50)	15(60)	9(81.8)	4(30.8)
	Papillary	0(0)	0(0)	0(0)	0(0)	0(0)	0(0)	8(61.5)
	Cylindrical	0(0)	0(0)	9(47.4)	10(50)	10(40)	2(28.2)	0(0)
Gland: stroma ratio	Increased	17(10)	74(63.8)	14(73.7)	12(60)	25(100)	11(100)	13(100)
	Normal	153(90)	42(36.2)	5(26.3)	8(40)	0(0)	0(0)	0(0)
Vascular dilation	Present	25(14.7)	92(79.3)	14(73.7)	18(90)	11(44)	3(27.3)	10(76.9)
	Absent	145(85.3)	24(20.7)	5(26.3)	2(10)	14(56)	8(72.7)	3(23.1)
Vascular congestion	Present	30(17.6)	85(73.3)	13(68.4)	16(80)	18(72)	10(90.9)	4(30.8)
	Absent	140(82.4	31(26.7	6(31.6)	4(20)	7(28)	1(9.1)	9(69.2)
Stromal hemorrhages	Present	14(8.2)	22(19.0)	10(52.6)	6(30)	17(68)	10(90.9)	4(30.8)
	Absent	156(91.8)	94(81)	9(47.4)	14(70)	89(32)	1(9.1)	9(69.2)
Mitotic score	1	153(90)	101(87.1)	15(78.9)	17(85)	4(16)	0(0)	0(0)
	2	17(10)	11(9.5)	4(21.1)	3(15)	17(68)	2(18.2)	0(0)
	3	0(0)	3(2.6)	0(0)	0(0)	4(16)	9(81.8)	0(0)
	4	0(0)	1(0.9)	0(0)	0(0)	0(0)	0(0)	10(76.9)
	5	0(0)	0(0)	0(0)	0(0)	0(0)	0(0)	3(23.1)
Total		170	116	19	20	25	11	13

Table 3 depicts the means of microvessel density, diameters, and height of glandular epithelium of all the six groups of histopathological diagnoses that were included in the present study after morphometric analysis.

**Table 3:** Means and standard deviations of various morphometric parameters in different histological groups PP: Proliferative phase, SP: Secretory phase, EP: Endometrial polyp, DPP: Disordered proliferative phase, HA: Hyperplasia without atypia, EIN: Endometrial intra epithelial neoplasia, EC: Endometrial carcinoma.

Histological diagnosis	n	Microvessel density (vessels/mm^2^) Mean±SD	Microvessel diameter (µm) Mean±SD	Height of glandular epithelium (µm) Mean±SD	Median mitotic figure score
PP	170	23.93±5.27	5.72±3.45	9.15±1.83	1
SP	116	23.03±5.77	7.82±4.56	9.12±2.24	1
DPP	19	21.42±5.93	33.42±2.83	48.93±5.54	1
EP	20	24.15±7.26	8.26±10.34	2.96±1.23	1
HA	25	21.08±3.82	70.67±14.86	42.33±6.89	2
EIN	11	28.36±1.80	83.7±24.56	135.63±14.78	3
EC	13	49.23±3.52	150±34.21	155.82±43.38	4

As evidenced in Table 4, on the statistical analysis of various parameters included in the morphometric analysis, it was found that the most useful parameters for differentiating various categories were mean vascular diameter and height of glandular epithelium. These parameters were significantly different in all groups (p value was less than or equal to 0.05) excepting a few. Mean vascular diameter was not significantly different in the secretory phase versus endometrial polyp and hyperplasia without atypia versus endometrial intra epithelial neoplasia (EIN) carcinoma (p-values 0.75 and 0.06, respectively). Height of glandular epithelium was not significantly different in proliferative versus secretory phase and EIN versus endometrial carcinoma (p value 0.9 and 0.16 respectively). When the other two parameters were studied, microvessel density and mitotic figure scores were found to be not very significantly different in various benign lesions. However, they were significantly higher in precursor and malignant lesions.

**Table 4:** Obtained p-values* for individual parameters when different histological groups are compared *Independent T-test was used to calculate p-values in SPSS software, version 24 (IBM Corp., Armonk, NY). PP: Proliferative phase, SP: Secretory phase, EP: Endometrial polyp, DPP: Disordered proliferative phase, HA: Hyperplasia without atypia, EIN: Endometrial intra epithelial neoplasia, EC: Endometrial carcinoma.

S.No	Comparison of various groups	Mean vascular density	Mean vascular diameter	Height of glandular epithelium	Mitotic figures score
1	PP Vs SP	0.19	<0.001	0.9	0.77
	PP Vs DP	0.06	<0.001	<0.001	0.92
	PP Vs EP	0.86	0.02	<0.001	1.00
	PP Vs HA	0.01	<0.001	<0.001	<0.001
	PP Vs EIN	0.006	<0.001	<0.001	<0.001
	PP Vs EC	<0.001	<0.001	<0.001	<0.001
2	SP Vs DP	0.25	<0.001	<0.001	1.00
	SP Vs EP	0.45	0.75	<0.001	1.00
	SP Vs HA	0.1	<0.001	<0.001	<0.001
	SP Vs EIN	0.003	<0.001	<0.001	<0.001
	SP Vs EC	<0.001	<0.001	<0.001	<0.001
3	DP Vs EP	0.19	<0.001	<0.001	1.00
	DP Vs HA	0.81	<0.001	0.001	<0.001
	DP Vs EIN	<0.001	<0.001	<0.001	<0.001
	DP Vs EC	<0.001	<0.001	<0.001	<0.001
4	EP Vs HA	0.07	<0.001	<0.001	<0.001
	EP Vs EIN	0.06	<0.001	<0.001	<0.001
	EP Vs EC	<0.001	<0.001	<0.001	<0.001
5	HA Vs EIN	<0.001	0.06	<0.001	<0.001
	HA Vs EC	<0.001	<0.001	<0.001	<0.001
6	EIN Vs EC	<0.001	<0.001	0.16	<0.001

## Discussion

The endometrium is an organ that undergoes changes in angiogenesis in a cyclical fashion. Angiogenesis in the endometrium under normal physiological conditions is a finely tuned process. It is controlled by a complex interplay of the microenvironment, pro-angiogenic and anti-angiogenic factors, as well as endogenous and exogenous hormones [3,4]. As evidenced in many studies, any imbalance of this mechanism can give rise to abnormal uterine bleeding [5-8]. Furthermore, in tumors that are normally prone to hypoxia, the balance in angiogenesis is further disrupted due to an increase in various pro-angiogenic factors like hypoxia-inducible factors. The present study is an attempt to study these structural alterations and morphology of vessels in various histological lesions of the endometrium.

As in other studies about AUB, [8] the most common histological pattern that was observed in the present study is the proliferative phase followed by the secretory phase and the least common lesion was endometrial carcinoma. In the current study, routinely processed, sectioned, and stained slides (Hematoxylin and Eosin stain) were used to do morphometric analysis. Being a routine operation, it was easy and swift. There are a few studies that have employed immunohistochemical markers like CD34, VEGF, factor VIII, actin, and CD31 for studying endothelial proliferation and its expression in endometrial stromal cells and glandular epithelium [9-11]. Hvingel et al. [9] and Mehra et al. [11], in their studies, showed that mean microvascular density and stromal expression of CD34 and actin were significantly higher in precursor and carcinoma lesions when compared to benign lesions.

In the present study, when proliferative and secretory patterns of endometrium were compared, it was found that there was not much of a difference in mitotic score, mean vascular density (Table 2), or height of glandular epithelium. However, mean vascular diameter was significantly higher in secretory phase. These findings are consistent with other studies [8]. This could be because angiogenesis in proliferative phase is mostly because of proliferation of endothelial cells and this is continued in secretory phase by elongation, coiling and intussusception of vessels with reduced proliferative activity of endothelial cells [3].

In the present study, vascular congestion, dilation, and the presence of stromal hemorrhages were more common in the precursor and carcinoma group than in the other groups. These results are similar to those obtained by the other groups [8]. Our findings can be substantiated by the fact that endometrial disorders are pathogenically associated with abnormal vasculature resulting in defective vascular remodelling and increased vascular fragility [3,11].

The method used for counting microvessel density in the present study is similar to that used by Makhija et al., [8]. However, instead of expressing it as a number per high power field, which varies based on the field number of the eyepiece used, an area of one square millimeter was used. This method helps attain uniformity in different setups. Our values are comparable with other studies [8,12,13].

Height of glandular epithelium was found to be lowest in endometrial polyp (2.96±1.23µm) and highest in endometrial carcinoma (155.82±43.38µm). Also it was found to be a useful parameter when comparing benign versus precursor or malignant group. However, there was no significant difference when comparing disordered proliferative endometrium versus hyperplasia without atypia or endometrial intraepithelial lesion versus endometrial carcinoma. Our findings are in correlation with those of Baak et al., who in their study, further highlighted the fact that this parameter can be used to prognosticate patients with hyperplasia and assess the risk for malignant transformation [14].

As presented in the current study, when various histological groups were compared for all the morphometric parameters individually, significant differences were observed in all studied parameters studied between the two groups at the extremes of the histological spectrum (proliferative or secretory versus endometrial carcinoma), however, while adjacent histological groups were compared (like disordered proliferative endometrium versus hyperplasia without atypia or endometrial polyp versus secretory/ proliferative endometrium, endometrial intra epithelial neoplasia versus endometrial carcinoma), use of more than one parameter is recommended.

Limitations of the present study include small sample size and selection bias (being a single centered retrospective study).

## Conclusions

As evidenced in the present study, endometrial glandular and blood vessel morphometry varies significantly in different endometrial lesions, and hence glandular and vascular morphometry can be used to differentiate various proliferative disorders of endometrium. Benign and malignant lesions showed significant differences in morphometry of endometrial glands and vasculature. This study also highlights the fact that abnormal angiogenesis forms the core of pathogenesis in AUB due to endometrial pathologies. In the current era of new anti angiogenic therapies, endometrial angiogenesis and changes in vascular morphology can be targeted and thus improve treatment modalities and patient care.
